# Integrated Bioinformatic Analysis of the Expression and Prognosis of Caveolae-Related Genes in Human Breast Cancer

**DOI:** 10.3389/fonc.2021.703501

**Published:** 2021-08-26

**Authors:** Yao Tian, Xiaofeng Liu, Jing Hu, Huan Zhang, Baichuan Wang, Yingxi Li, Li Fu, Ran Su, Yue Yu

**Affiliations:** ^1^The First Department of Breast Cancer, Tianjin Medical University Cancer Institute and Hospital, Tianjin, China; ^2^Key Laboratory of Breast Cancer Prevention and Therapy, Ministry of Education, Tianjin Medical University Cancer Institute and Hospital, National Clinical Research Center for Cancer, Tianjin, China; ^3^Key Laboratory of Cancer Prevention and Therapy, Tianjin’s Clinical Research Center for Cancer, Tianjin Medical University Cancer Institute and Hospital, Tianjin, China; ^4^Department of General Surgery, Tianjin Medical University General Hospital, Tianjin, China; ^5^Anhui Medical University Clinical College of Chest, Hefei, China; ^6^Department of Thoracic Surgery, Anhui Chest Hospital, Hefei, China; ^7^Department of Pathogen Biology, School of Basic Medical Sciences, Tianjin Medical University, Tianjin, China; ^8^Department of Breast Cancer Pathology and Research Laboratory, Tianjin Medical University Cancer Institute and Hospital, Tianjin, China; ^9^School of Computer Software, College of Intelligence and Computing, Tianjin University, Tianjin, China

**Keywords:** CAVs, CAVINs, breast cancer, biomarkers, bioinformatics, prognosis

## Abstract

Caveolae-related genes, including CAVs that encodes caveolins and CAVINs that encodes caveolae-associated proteins cavins, have been identified for playing significant roles in a variety of biological processes including cholesterol transport and signal transduction, but evidences related to tumorigenesis and cancer progression are not abundant to correlate with clinical characteristics and prognosis of patients with cancer. In this study, we investigated the expression of these genes at transcriptional and translational levels in patients with breast cancer using Oncomine, Gene Expression Profiling Interactive Analysis (GEPIA), cBioPortal databases, and immunohistochemistry of the patients in our hospital. Prognosis of patients with breast cancer based on the expressions of CAVs and CAVINs was summarized using Kaplan-Meier Plotter with their correlation to different subtyping. The relevant molecular pathways of these genes were further analyzed using Kyoto Encyclopedia of Genes and Genomes (KEGG) pathway database and Gene Set Enrichment Analysis (GSEA). Results elucidated that expression levels of CAV1, CAV2, CAVIN1, CAVIN2, and CAVIN3 were significantly lower in breast cancer tissues than in normal samples, while the expression level of CAVIN2 was correlated with advanced tumor stage. Furthermore, investigations on survival of patients with breast cancer indicated outstanding associations between prognosis and CAVIN2 levels, especially for the patients with estrogen receptor positive (ER+) breast cancer. In conclusion, our investigation indicated CAVIN2 is a potential therapeutic target for patients with ER+ breast cancer, which may relate to functions of cancer cell surface receptors and adhesion molecules.

## Introduction

Caveolae are cave-shaped invaginated structures of cell plasma membrane at the range of 50–100 nm (1). They exist in many cell types and are enriched in adipocytes and endothelial cells (2, 3). Within caveolae, caveolins and cavins (caveolae-associated proteins) consist of the key components that involve in a variety of biological processes including signal transduction, endocytosis, cell cycle regulation, and apoptosis (4). It has been reported that the functions of caveolins and cavins are relevant to the progression of malignant tumor, such as prostate cancer (5, 6), breast cancer (7–10), lung cancer (11), liver cancer (12), kidney cancer (13), colon cancer (14), and pancreatic cancer (15, 16).

Breast cancer is the most common cancer in women worldwide, contributing 11.7% of the total number of new cancer cases diagnosed in 2020 (17). On the basis of traditional therapeutic methods such as surgery, chemotherapy and radiotherapy, the introduction of molecular subtyping has brought more significant improvement to the precise treatment for breast cancer. It is mainly divided into four subtypes based on the expression of estrogen receptor (ER), progesterone receptor (PR), and human epidermal growth factor receptor 2 (HER2): Luminal A (ER+ and/or PR+ and HER2-), Luminal B (ER+ and/or PR+ and HER2+), HER2 enriched (ER-, PR-, and HER2+), and basal-like subtype (triple-negative of the above receptors) (18). Patient with Luminal A or B breast cancer can benefit from endocrine therapy by inhibiting ER. Development of anticancer drugs targeting HER2 has also brought better outcomes to those HER2 positives (19). Although the application of novel and advanced treatment has significantly improved the survival of patients with breast cancer, there are still some individual developed distant metastasis at early stage. Meanwhile, the therapeutic effect of triple negative breast cancer has not been significantly improved due to the lack of specific molecular targets (20). Therefore, it is important to identify molecular targets related to the occurrence, progression, metastasis, and prognosis of breast cancer through the existing research data.

Currently, there are seven family members found as caveolins and cavins. They are caveolin-1 (CAV1), caveolin-2 (CAV2), caveolin-3 (CAV3) (21), cavin-1 (CAVIN1, known as polymerase-1 and transcript release factor, PTRF), cavin-2 (CAVIN2, known as serum deprivation protein response, SDPR), cavin-3 (CAVIN3, known as sdr-related gene product that binds to c-kinase, SRBC), and cavin-4 (CAVIN4, known as muscle-restricted coiled-coil protein, MURC). Caveolins usually play a paradoxical role in the development of diseases, while they are implicated in both tumor suppression and oncogenesis (22, 23). As the principal structural component of caveolae membrane, the ablation of caveolin-1 can induce caveolae loss (24). Caveolin-1 was also reported to associate with breast cancer stem cell enrichment (25). Kang et al. have showed caveolin-1 expression level was a potential indicator for predicting efficacy of docetaxel in the treatment of triple-negative breast cancer (26). Caveolin-2 can directly interact with caveolin-1 (27). Shatseva et al. have reported miR-199a-3p can promote breast cancer cell proliferation through inhibiting caveolin-2 (28), which indicated caveolin-2 as a tumor suppressor gene. It was also necessary for estrogen-dependent breast cancer cell proliferation. Caveolin-3 is predominantly expressed in muscle cells (29). Few studies reported the function caveolin-3 in cancer. Nevertheless, the functions of caveolins in cancer are still under intensive investigations.

Cavins are caveolae-associated proteins and are indispensable for caveolae biogenesis. Similar to caveolins, cavins also have prominently differential tissue distributions. As a resident protein in caveolae, cavin-1 is widely expressed in a variety types of tissues (30). Yi et al. has reported cavin-1 was essential for drug resistance in breast cancer cell (31). Loss of cavin-1 is also accompanied by destruction of caveolae (32). Cavin-2 is about 20% structurally similar to cavin-1, but the alteration in expression of cavin-2 does not influence the number of caveolae (33). It has been reported to play significant roles in inhibiting cancer cell migration and metastatic potentials when overexpressed (34). A previous study of our group has discovered that cavin-2 depletion can induce epithelial-mesenchymal transition in breast cancer cells by activating TGF-β signaling pathway (10). Cavin-3 is reported to be relevant to CAV1 during caveolae budding (35). Several studies have emphasized the epigenetic modification of cavin-3 can contribute to the pathogenesis of cancer (36, 37), indicating it as a tumor suppressor candidate. Cavin-4 is only abundant in muscle cells and is able to interact with cavin-2. Faggi et al. has reported cavin-4 is important in the differentiation process of rhabdomyosarcoma in combination with caveolin-3 (38).

To comprehensively investigate how the dysregulation of CAVs and CAVINs levels associate with clinical characteristics of patients with breast cancer, in this study we conducted integrated bioinformatic analysis using online database and analytic tools to explore whether CAVs and CAVINs are relevant to the prognosis of patients with breast cancer and other neighboring signaling pathways involved. Protein level expression of CAVs and CAVINs were validated using real-world immunohistochemistry samples. These results will provide solid evidences for the prediction of prognosis and precise therapy towards breast carcinoma by targeting caveolae-related genes.

## Materials and Methods

### Ethical Statement

Benchwork study using tissue samples from patients with breast cancer was approved by the Institutional Review Board of Tianjin Medical University Cancer Institute and Hospital. Relevant investigations were conducted under the principles stated in the Declaration of Helsinki. Studies using datasets were accomplished and retrieved from publications, therefore relevant ethical documents were considered as obtained or approved.

### Oncomine Analysis

We used Oncomine gene expression array datasets (https://www.oncomine.org/resource/login.html) to analyze the transcriptional levels of caveolae-related proteins in different types of cancer. The mRNA levels of CAVs and CAVINs in cancer samples from patients were compared with those in normal samples using a Student’s t test to generate a *p* value. Recommended cutoffs of *p* value and fold change were defined as 0.01 and 2 respectively.

### GEPIA Dataset

We utilized GEPIA (http://gepia.cancer-pku.cn) to further investigate the correlation of gene expression based on RNA sequencing and tissue type or clinical stages of the patients with breast cancer, which is a novel web tool for analyzing RNA-seq data in 9,736 tumors and 8,587 normal samples from the The Cancer Genome Atlas (TCGA) and the Genotype-Tissue Expression (GTEx) projects (39). The individualized studies were conducted under standard processing requirements.

### Immunohistochemistry

Formalin-fixed, paraffin-embedded (FFPE) sections of breast cancer tissues and paired normal samples were subjected to immunostaining with a rabbit monoclonal antibody of CAVs or CAVINs (all from Abcam, UK. Caveolin-1[CAV1], ab32577; Caveolin-2 [CAV2], ab79397; Caveolin-3 [CAV3], ab173575; PTRF [CAVIN1], ab48824; SDPR [CAVIN2], ab76867; SRBC [CAVIN3], ab179923; MURC [CAVIN4], ab121647). In brief, 5-μm thick tissue sections were deparaffinized, rehydrated and subjected to antigen retrieval by boiling in sodium citrate buffer (10 mM, pH 6.0). The sections were incubated at 4°C overnight with above antibodies at 1:100 dilution, and then exposed with HRP-conjugated secondary antibody at 1:500 dilution (Abcam, UK) followed by covering 3,3’-diaminobenzidine (DAB) (Sigma, USA). After mounted, the slides were visualized under a light microscope (Carl Zeiss, Germany) and images were captured by microscopy camera (Carl Zeiss, Germany).

To quantitatively evaluate the expression levels of each protein in the samples from patients with breast cancer, we calculated percentage of cells stained and the staining intensity, and scored the IHC images using the following criteria: (a) percentage of stained cells: 0 (0%), 1 (1%–25%), 2 (26%–50%), 3 (51%–75%), and 4 (> 75%); and (b) staining intensity: 0 (negative staining), 1 (weak staining), 2 (moderate staining), and 3 (strong staining). The multiplier of the scores (0-12) was summarized as scatter plot.

### The Cancer Genome Atlas, cBioPortal, GeneMANIA, and STRING

The Cancer Genome Atlas (TCGA) database has sequencing, clinical information, and pathological results of patients with different types of cancer (40). We used cBioPortal (https://www.cbioportal.org/results/oncoprint?session_id=5e623de7e4b0ff7ef5fd9620) to further analyze the data from 1,108 cases of breast invasive carcinoma with complete pathological reports in TCGA. Genomic profiles of CAVs and CAVINs were investigated, including mutations, copy number alterations (CNAs) from genomic identification of significant targets in cancer (GISTIC), mRNA expression Z scores (RNA-seq v.2 RSEM), and protein expression Z scores (reverse phase protein array [RPPA]). Co-expression and network were determined according to the online instructions of cBioPortal. The protein-protein interaction networks of CAVs and CAVINs were constructed using the online database Gene MANIA (https://genemania.org/) and STRING (https://string-db.org).

### The Kaplan-Meier Plotter

The online database Kaplan–Meier plotter (http://kmplot.com/analysis/) was used to investigate the prognosis of certain mRNA expression according to gene expression data and survival information of patients with breast cancer (http://kmplot.com/analysis/index.php?p=service&cancer= breasthttp://kmplot.com/analysis/index.php?p=service&cancer=%20breast) (41). To analyze overall survival (OS) and relapse-free survival (RFS) of the patients, the samples were divided into high expression group and low expression group by the median expression level of the gene. The risk ratio (hazard ratio [HR]) with 95% confidence interval (CI) and log rank *p* value for each predictor were determined to generate Kaplan-Meier plots. In this study, only the probe sets with best JetSet scores for CAVs and CAVINs were selected to generate Kaplan-Meier plots. Number of members was displayed below the main plot.

### Gene Set Enrichment Analysis

Gene Set Enrichment Analysis (GSEA, https://www.gsea-msigdb.org/) is a computational method that determines whether a priory defined set of genes shows statistically significant, concordant differences between different phenotypes. GSEA was used to explore pathways and gene sets associated with CAVIN2 in breast cancer. Gene expression profiles of 406 breast cancer samples were downloaded from TCGA dataset. According to the order of expression level of CAVIN2 and the prognosis of cases, the optimal threshold in the ROC curve was divided into HIGH expression group and LOW expression group. GSEA v4.0.3 was used to determine whether the members of the gene set from the MSigDB database are randomly distributed at the top or bottom of the ranking. If most members of a gene set were positively related to the HIGH group, the set was termed associated with LOW group.

## Results

### Comparison of Transcriptional Levels of CAVs and CAVINs in Patients With Breast Cancer

**Figure 1:**
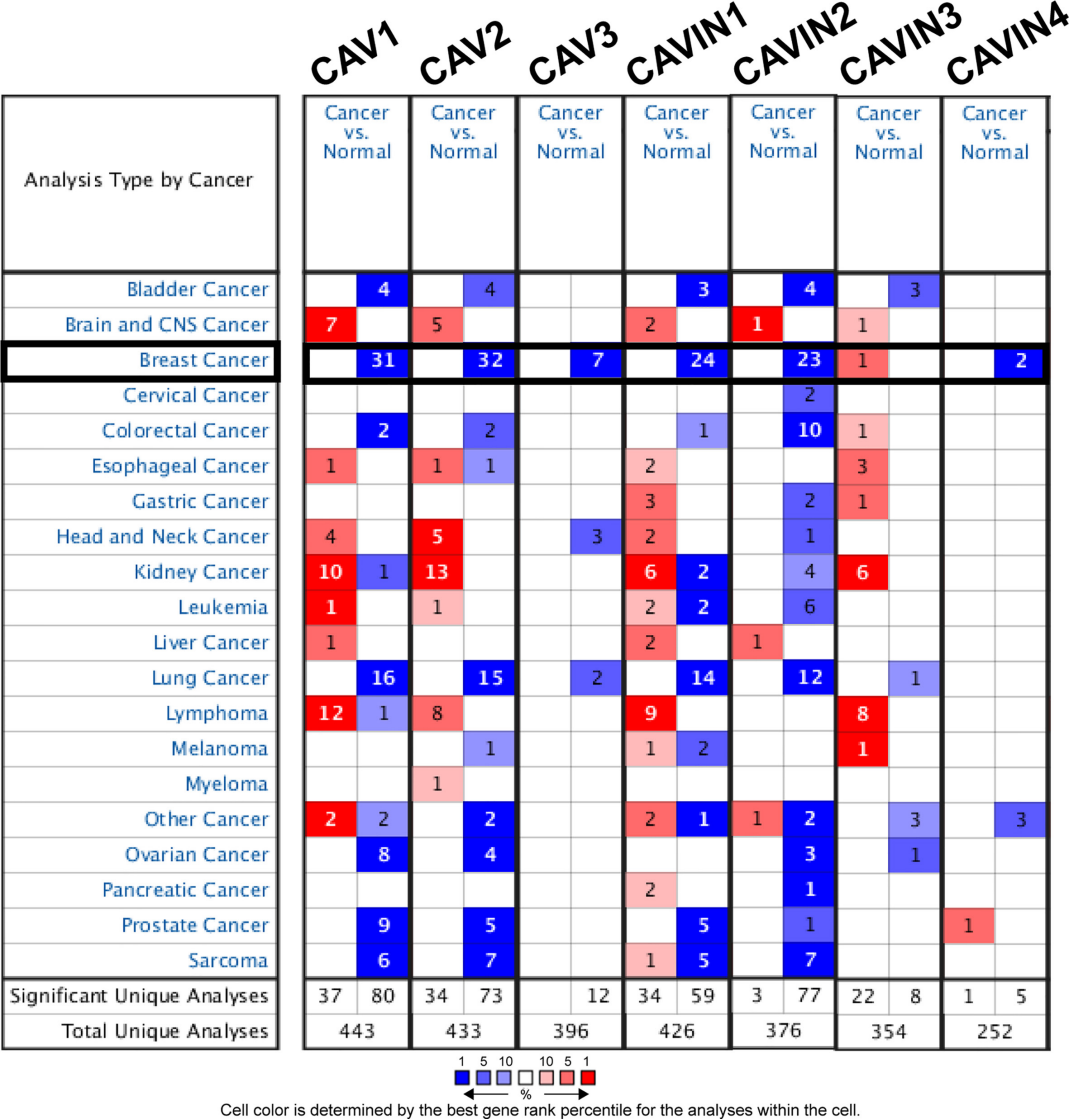
Comparison of transcriptional levels of CAVs and CAVINs in different types of cancers.

As three CAV proteins and four CAVIN proteins commonly exist in human cells, we firstly compared the transcriptional levels of CAVs and CAVINs in different types of cancer with those in normal tissues using Oncomine database (**Figure 1**). The expression levels of CAV1 mRNA were significantly downregulated in patients with breast cancer in 31 datasets. In both Sorlie Breast statistics (18) and Sorlie Breast 2 statistics (42), the lower expression of CAV1 was most prominent in fibroadenoma, with a fold change of -14.128 and -12.864 respectively (**Table 1**). In TCGA breast statistics (40), Curtis Breast Statistics (43), Richardson Breast 2 Statistics (44), and Perou Breast Statistics (45), CAV1 was also less expressed in cancer tissues than in normal tissues, but the most prominent value appeared in ductal breast carcinoma, with a fold change of -11.297, -7.821, -8.398, and -9.284 respectively (**Table 1**). CAV2 mRNA was also found to be lower expressed in cancer tissues in 32 datasets (**Figure 1**). The most significant fold changes existed in ductal breast carcinoma in most of the datasets except TCGA, which revealed the most significant differences in intraductal cribriform breast adenocarcinoma with a fold change of -11.089 (**Table 1**). TCGA breast statistics have also showed CAV3 were lower expressed in cancer tissues, with the most prominent fold change of -11.604 in intraductal cribriform breast adenocarcinoma (**Table 1**).

**Table 1:** Significant expression changes of caveolae-related proteins in transcription levels between different types of breast cancer and normal breast tissues (Oncomine Database).

	Type of Breast Cancer *versus* Normal Breast Tissue	Fold Change	p Value	t Test	Source and/or Reference	
CAV1	Ductal Breast Carcinoma	-5.462	9.14E-27	-17.953	Sorlie Breast 2 Statistics	PMID:12829800
	Fibroadenoma	-12.864	4.72E-04	-6.958	Sorlie Breast 2 Statistics	
	Lobular Breast Carcinoma	-6.906	3.00E-03	-3.655	Sorlie Breast 2 Statistics	
	Fibroadenoma	-14.128	5.10E-04	-6.939	Sorlie Breast Statistics	PMID:11553815
	Ductal Breast Carcinoma	-5.473	2.01E-06	-10.028	Sorlie Breast Statistics	
	Lobular Breast Carcinoma	-12.424	7.00E-03	-3.94	Sorlie Breast Statistics	
	Invasive Breast Carcinoma	-7.607	1.92E-35	-17.06	TCGA Breast Statistics	TCGA
	Invasive Lobular Breast Carcinoma	-6.366	8.58E-20	-12.522	TCGA Breast Statistics	
	Mixed Lobular and Ductal Breast Carcinoma	-4.73	8.31E-12	-10.033	TCGA Breast Statistics	
	Invasive Ductal Breast Carcinoma	-11.297	1.00E-40	-26.387	TCGA Breast Statistics	
	Mucinous Breast Carcinoma	-5.711	7.32E-06	-9.074	TCGA Breast Statistics	
	Invasive Ductal and Lobular Carcinoma	-8.837	9.05E-05	-12.806	TCGA Breast Statistics	
	Male Breast Carcinoma	-8.963	1.14E-04	-12.654	TCGA Breast Statistics	
	Intraductal Cribriform Breast Adenocarcinoma	-5.777	1.00E-03	-7.544	TCGA Breast Statistics	
	Mucinous Breast Carcinoma	-4.257	3.94E-37	-17.398	Curtis Breast Statistics	PMID:22522925
	Tubular Breast Carcinoma	-3.784	6.91E-45	-18.091	Curtis Breast Statistics	
	Invasive Lobular Breast Carcinoma	-4.9	2.23E-60	-21.145	Curtis Breast Statistics	
	Invasive Ductal and Invasive Lobular Breast Carcinoma	-5.4	1.68E-48	-20.446	Curtis Breast Statistics	
	Invasive Ductal Breast Carcinoma	-7.821	8.68E-91	-41.04	Curtis Breast Statistics	
	Medullary Breast Carcinoma	-4.592	6.01E-28	-16.472	Curtis Breast Statistics	
	Breast Carcinoma	-4.565	8.39E-13	-13.211	Curtis Breast Statistics	
	Invasive Breast Carcinoma	-4.065	1.17E-12	-10.928	Curtis Breast Statistics	
	Benign Breast Neoplasm	-2.73	2.84E-04	-10.894	Curtis Breast Statistics	
	Ductal Breast Carcinoma *In situ*	-3.55	5.57E-06	-7.332	Curtis Breast Statistics	
	Breast Phyllodes Tumor	-4.268	6.00E-03	-4.352	Curtis Breast Statistics	
	Invasive Breast Carcinoma Stroma	-31.965	2.62E-31	-23.515	Finak Breast Statistics	PMID:18438415
	Invasive Ductal Breast Carcinoma Stroma	-2.077	5.01E-05	-5.808	Ma Breast 4 Statistics	PMID:19187537
	Ductal Breast Carcinoma *In situ* Epithelia	-4.516	1.28E-05	-5.477	Ma Breast 4 Statistics	
	Invasive Ductal Breast Carcinoma Epithelia	-3.683	8.00E-03	-2.759	Ma Breast 4 Statistics	
	Ductal Breast Carcinoma	-8.398	2.42E-09	-10.429	Richardson Breast 2 Statistics	PMID:16473279
	Ductal Breast Carcinoma	-9.284	5.00E-03	-7.084	Perou Breast Statistics	PMID:10963602
CAV2	Fibroadenoma	-5.489	1.00E-03	-6.986	Sorlie Breast Statistics	PMID:11553815
	Lobular Breast Carcinoma	-5.038	5.00E-03	-4.514	Sorlie Breast Statistics	
	Ductal Breast Carcinoma	-5.714	1.00E-03	-7.357	Sorlie Breast Statistics	
	Invasive Lobular Breast Carcinoma	-4.797	1.12E-19	-12.538	TCGA Breast Statistics	TCGA
	Mixed Lobular and Ductal Breast Carcinoma	-6.152	8.77E-12	-12.276	TCGA Breast Statistics	
	Invasive Breast Carcinoma	-4.587	1.19E-30	-14.97	TCGA Breast Statistics	
	Invasive Ductal Breast Carcinoma	-7.006	3.54E-41	-24.744	TCGA Breast Statistics	
	Mucinous Breast Carcinoma	-9.574	2.47E-05	-13.146	TCGA Breast Statistics	
	Male Breast Carcinoma	-6.681	2.33E-05	-11.408	TCGA Breast Statistics	
	Intraductal Cribriform Breast Adenocarcinoma	-11.089	3.00E-03	-7.953	TCGA Breast Statistics	
	Invasive Ductal and Lobular Carcinoma	-7.218	2.00E-03	-7.867	TCGA Breast Statistics	
	Fibroadenoma	-4.998	1.00E-03	-6.617	Sorlie Breast 2 Statistics	PMID:12829800
	Lobular Breast Carcinoma	-4.287	1.00E-03	-4.723	Sorlie Breast 2 Statistics	
	Ductal Breast Carcinoma	-5.787	1.00E-03	-7.653	Sorlie Breast 2 Statistics	
	Invasive Ductal and Invasive Lobular Breast Carcinoma	-4.241	1.92E-51	-20.149	Curtis Breast Statistics	PMID:22522925
	Breast Carcinoma	-2.111	1.04E-17	-13.168	Curtis Breast Statistics	
	Mucinous Breast Carcinoma	-2.069	8.17E-35	-15.247	Curtis Breast Statistics	
	Ductal Breast Carcinoma *In situ*	-2.025	5.38E-10	-10.403	Curtis Breast Statistics	
	Tubular Breast Carcinoma	-3.93	5.76E-43	-18.687	Curtis Breast Statistics	
	Invasive Breast Carcinoma	-2.036	2.87E-15	-11.012	Curtis Breast Statistics	
	Invasive Lobular Breast Carcinoma	-3.761	3.90E-55	-19.555	Curtis Breast Statistics	
	Medullary Breast Carcinoma	-2.1	2.57E-24	-13.222	Curtis Breast Statistics	
	Invasive Ductal Breast Carcinoma	-5.178	4.42E-73	-32.597	Curtis Breast Statistics	
	Breast Phyllodes Tumor	-3.494	3.67E-04	-7.126	Curtis Breast Statistics	
	Invasive Mixed Breast Carcinoma	-3.272	2.00E-03	-4.346	Radvanyi Breast Statistics	PMID:16043716
	Invasive Lobular Breast Carcinoma	-2.624	5.00E-03	-3.207	Radvanyi Breast Statistics	
	Invasive Ductal Breast Carcinoma	-2.346	8.00E-03	-2.888	Radvanyi Breast Statistics	
	Lobular Breast Carcinoma	-3.759	3.00E-03	-4.583	Perou Breast Statistics	PMID:10963602
	Ductal Breast Carcinoma	-5.945	1.00E-03	-9.231	Perou Breast Statistics	
	Ductal Breast Carcinoma	-5.031	6.00E-10	-9.235	Richardson Breast 2 Statistics	PMID:16473279
	Invasive Breast Carcinoma	-10.091	1.44E-04	-13.884	Glück Breast Statistics	PMID:21373875
	Invasive Ductal Breast Carcinoma Epithelia	-4.693	7.00E-03	-2.961	Ma Breast 4 Statistics	PMID:19187537
CAV3	Invasive Lobular Breast Carcinoma	-4.6	2.34E-22	-12.958	TCGA Breast Statistics	TCGA
	Invasive Breast Carcinoma	-5.762	6.70E-34	-16.936	TCGA Breast Statistics	
	Mixed Lobular and Ductal Breast Carcinoma	-5.692	1.41E-10	-13.479	TCGA Breast Statistics	
	Invasive Ductal Breast Carcinoma	-7.809	2.49E-36	-24.176	TCGA Breast Statistics	
	Mucinous Breast Carcinoma	-6.402	1.42E-04	-9.903	TCGA Breast Statistics	
	Invasive Ductal and Lobular Carcinoma	-7.293	1.38E-04	-12.868	TCGA Breast Statistics	
	Intraductal Cribriform Breast Adenocarcinoma	-11.604	5.00E-03	-7.772	TCGA Breast Statistics	
Cavin-1/PTRF	Invasive Ductal Breast Carcinoma	-2.739	1.03E-122	-42.749	Curtis Breast Statistics	PMID:22522925
	Invasive Lobular Breast Carcinoma	-2.46	6.01E-46	-18.531	Curtis Breast Statistics	
	Invasive Ductal and Invasive Lobular Breast Carcinoma	-2.628	1.67E-33	-17.036	Curtis Breast Statistics	
	Medullary Breast Carcinoma	-3.31	4.20E-13	-11.173	Curtis Breast Statistics	
	Mucinous Breast Carcinoma	-3.539	1.09E-18	-13.639	Curtis Breast Statistics	
	Breast Carcinoma	-3.23	7.98E-07	-8.061	Curtis Breast Statistics	
	Tubular Breast Carcinoma	-2.23	3.64E-25	-14.495	Curtis Breast Statistics	
	Ductal Breast Carcinoma *In situ*	-2.645	3.36E-04	-5.023	Curtis Breast Statistics	
	Invasive Breast Carcinoma	-2.764	2.26E-05	-5.155	Curtis Breast Statistics	
	Ductal Breast Carcinoma	-4.015	1.86E-06	-11.704	Perou Breast Statistics	PMID:10963602
	Lobular Breast Carcinoma	-2.784	8.00E-03	-4.216	Perou Breast Statistics	
	Invasive Ductal Breast Carcinoma	-2.362	1.59E-09	-8.105	Zhao Breast Statistics	PMID:15034139
	Ductal Breast Carcinoma	-5.807	1.24E-11	-12.161	Richardson Breast 2 Statistics	PMID:16473279
	Ductal Breast Carcinoma *In situ* Epithelia	-5.638	1.26E-06	-8.205	Ma Breast 4 Statistics	PMID:19187537
	Lobular Breast Carcinoma	-2.974	9.00E-03	-3.765	Sorlie Breast Statistics	PMID:11553815
	Ductal Breast Carcinoma	-4.216	7.82E-05	-10.649	Sorlie Breast Statistics	
	Lobular Breast Carcinoma	-2.334	3.00E-03	-3.551	Sorlie Breast 2 Statistics	PMID:12829800
	Ductal Breast Carcinoma	-4.014	1.07E-04	-11.117	Sorlie Breast 2 Statistics	
	Intraductal Cribriform Breast Adenocarcinoma	-3.105	7.68E-12	-10.555	TCGA Breast Statistics	TCGA
	Mucinous Breast Carcinoma	-2.357	3.59E-07	-7.073	TCGA Breast Statistics	
	Invasive Breast Carcinoma	-3.21	6.66E-21	-11.201	TCGA Breast Statistics	
	Mixed Lobular and Ductal Breast Carcinoma	-2.766	7.52E-06	-6.982	TCGA Breast Statistics	
	Invasive Lobular Breast Carcinoma	-2.862	1.14E-12	-8.111	TCGA Breast Statistics	
	Invasive Ductal Breast Carcinoma	-4.04	1.48E-27	-16.278	TCGA Breast Statistics	
Cavin-2/SDPR	Invasive Lobular Breast Carcinoma	-4.857	4.64E-81	-27.075	Curtis Breast Statistics	PMID:22522925
	Tubular Breast Carcinoma	-5.474	6.55E-64	-26.805	Curtis Breast Statistics	
	Medullary Breast Carcinoma	-8.162	3.28E-56	-33.594	Curtis Breast Statistics	
	Invasive Ductal and Invasive Lobular Breast Carcinoma	-5.102	3.45E-63	-24.597	Curtis Breast Statistics	
	Invasive Ductal Breast Carcinoma	-7.136	6.27E-90	-44.083	Curtis Breast Statistics	
	Mucinous Breast Carcinoma	-5.711	1.73E-31	-19.506	Curtis Breast Statistics	
	Breast Carcinoma	-6.516	1.35E-13	-17.841	Curtis Breast Statistics	
	Invasive Breast Carcinoma	-6.158	6.91E-13	-13.096	Curtis Breast Statistics	
	Ductal Breast Carcinoma *In situ*	-4.993	1.93E-07	-10.85	Curtis Breast Statistics	
	Breast Phyllodes Tumor	-3.766	6.00E-03	-4.255	Curtis Breast Statistics	
	Invasive Ductal Breast Carcinoma Stroma	-11.593	5.95E-07	-7.798	Ma Breast 4 Statistics	PMID:19187537
	Ductal Breast Carcinoma *In situ* Epithelia	-2.864	4.81E-05	-5.169	Ma Breast 4 Statistics	
	Ductal Breast Carcinoma *In situ* Stroma	-3.589	5.31E-04	-3.914	Ma Breast 4 Statistics	
	Invasive Breast Carcinoma	-14.278	3.70E-33	-16.431	TCGA Breast Statistics	TCGA
	Mucinous Breast Carcinoma	-24.174	2.32E-14	-23.228	TCGA Breast Statistics	
	Invasive Ductal Breast Carcinoma	-25.347	2.29E-55	-32.344	TCGA Breast Statistics	
	Invasive Lobular Breast Carcinoma	-10.124	1.16E-17	-12.301	TCGA Breast Statistics	
	Mixed Lobular and Ductal Breast Carcinoma	-15.494	1.28E-05	-8.897	TCGA Breast Statistics	
	Intraductal Cribriform Breast Adenocarcinoma	-60.805	1.00E-03	-12.429	TCGA Breast Statistics	
	Invasive Ductal and Lobular Carcinoma	-12.004	3.00E-03	-9.072	TCGA Breast Statistics	
	Invasive Breast Carcinoma	-18.248	8.84E-06	-17.025	Glück Breast Statistics	PMID:21373875
	Invasive Ductal Breast Carcinoma	-6.155	2.00E-03	-3.749	Radvanyi Breast Statistics	PMID:16043716
	Ductal Breast Carcinoma	-4.895	1.43E-04	-6.997	Richardson Breast 2 Statistics	PMID:16473279
Cavin-3/SRBC/PRKCDBP	Invasive Breast Carcinoma Stroma	3.273	3.83E-16	13.421	Finak Breast Statistics	PMID:18438415
Cavin-4/MURC	Invasive Breast Carcinoma	-3.231	1.28E-10	-18.542	Glück Breast Statistics	PMID:21373875
	Invasive Ductal Breast Carcinoma	-2.592	7.39E-04	-4.158	Radvanyi Breast Statistics	PMID:16043716

In terms of CAVIN family, 24 datasets demonstrated lower expression of CAVIN1 in cancer tissues (**Figure 1**). Perou et al, Zhao et al (46), Richardson et al, Ma et al (47), Sorlie et al, and TCGA all presented the most significant differences in ductal breast cancer (**Table 1**). The evidences of CAVIN2 lower expression in cancer tissue were provide by 23 datasets (**Figure 1**), with Ma et al., Radvanyi et al. (48), and Richardson et al. showing the most significant differences in ductal breast cancer. In TCGA, the most prominent downregulation of CAVIN2 existed in intraductal cribriform breast adenocarcinoma with a fold change of -60.805, which was a similar to the situation of CAV2 and CAV3 (**Table 1**). CAVIN3 was found higher expressed in invasive breast carcinoma stroma only by Finak et al (49), with a fold change of 3.273. CAVIN4 was found lower expressed by Glück et al (50) and Radvanyi et al, with a fold change of -3.231 in invasive breast cancer and -2.592 in invasive ductal breast cancer respectively.

### Relationship Between the Transcriptional and Translational Levels of Caveolae-Related Proteins and Clinicopathological Characteristics of Patients With Breast Cancer

**Figure 2:**
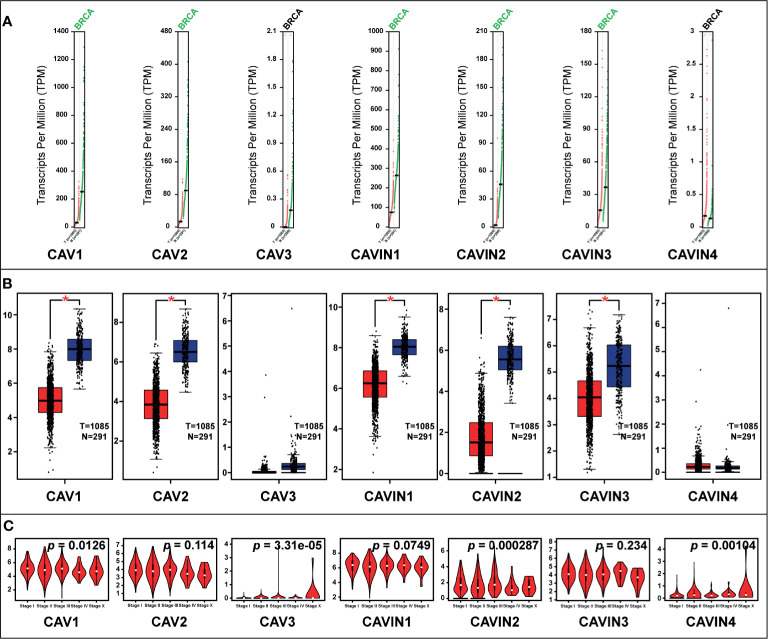
**(A)** Scatter diagram of expressions of CAVs and CAVINs in breast cancer. **(B)** Box plot of expression of CAVs and CAVINs in breast cancer (*p < 0.05). **(C)** Correlation between CAVs and CAVINs levels and clinical stages of patients with breast cancer.

We then compared the mRNA levels of CAVs and CAVINs in breast cancer tissues and normal ones using the GEPIA (Gene Expression Profiling Interactive Analysis) dataset (39). The results elucidated that the transcriptional levels of CAV1, CAV2, CAVIN1, CAVIN2, and CAVIN3 were significantly lower in breast cancer tissues than in normal samples (**Figures 2A, B**). However, it should be noticed that the expression levels of CAV3 and CAVIN4 in both cancer tissues and normal tissues were rather low (**Figure 2B**), indicating a limited role of these two proteins in the tumorigenesis and progression of breast cancer. Furthermore, the expression levels of these caveolae-related genes were further correlated with cancer stage of the patients. We found that CAV3, CAVIN2, and CAVIN4 were significantly differed among stages, while others did not show differences (**Figure 2C**).

**Figure 3:**
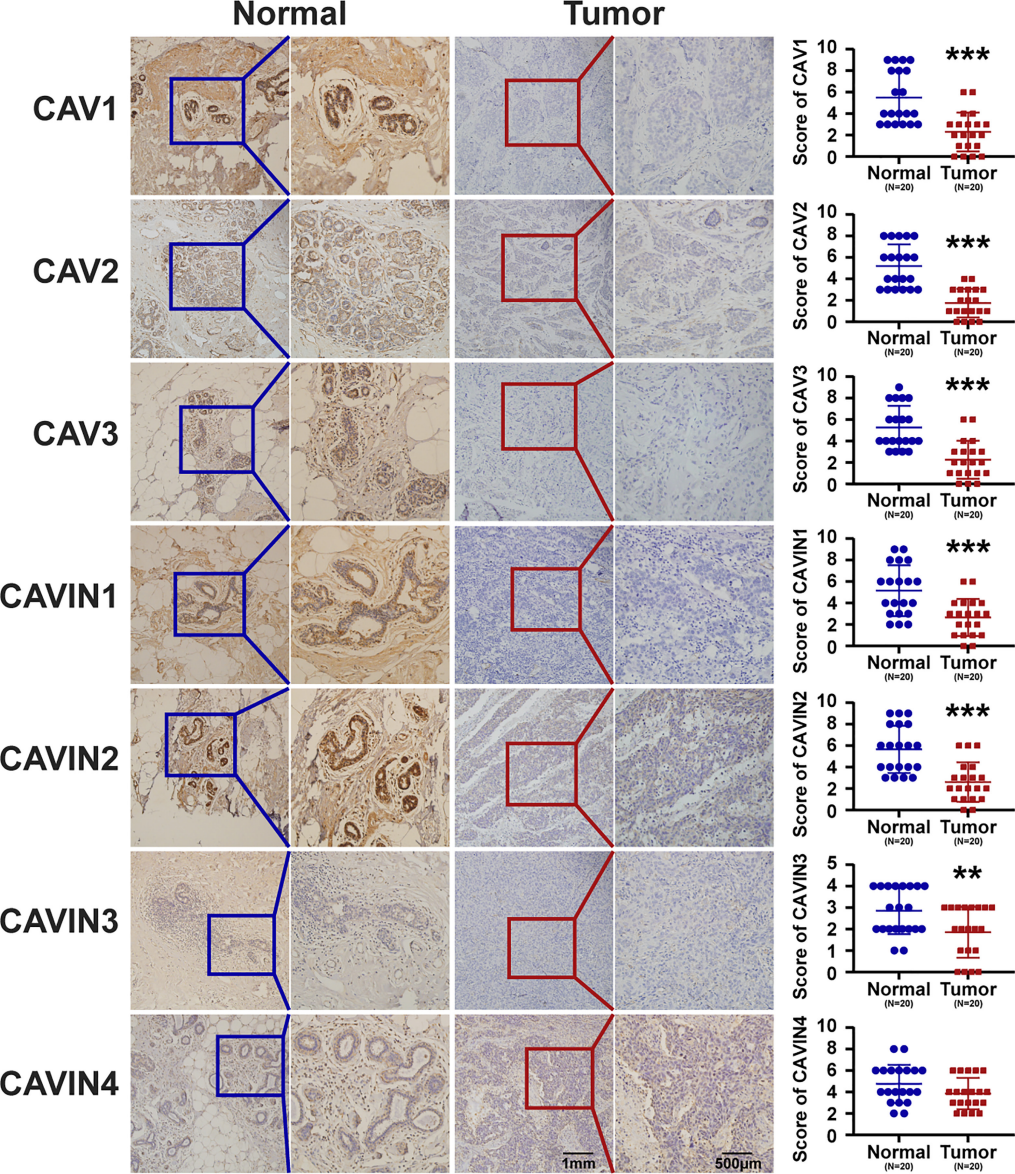
Representative immunohistochemical images of CAVs and CAVINs in cancer tissues with paired normal samples from patients with breast cancer. 20 samples were involved for IHC analysis and the corresponding IHC scores were summarized as scatter plots on the right. (**p < 0.01, ***p < 0.005).

To validate the results from database, we have conducted immunohistochemistry (IHC) to verify the expression status of CAVs and CAVINs in breast cancer tissues and paired normal samples from patients in our hospital. 20 paired samples from patients with breast cancer were involved in IHC experiments. The statistical results showed that CAV1, CAV2, CAV3, CAVIN1, and CAVIN2 were significantly less expressed in breast cancer tissues than in normal ones (**Figure 3**). Taken together, differences in expression level of CAVIN2 was the most prominent of all the caveolae-related genes.

**Figure 4:**
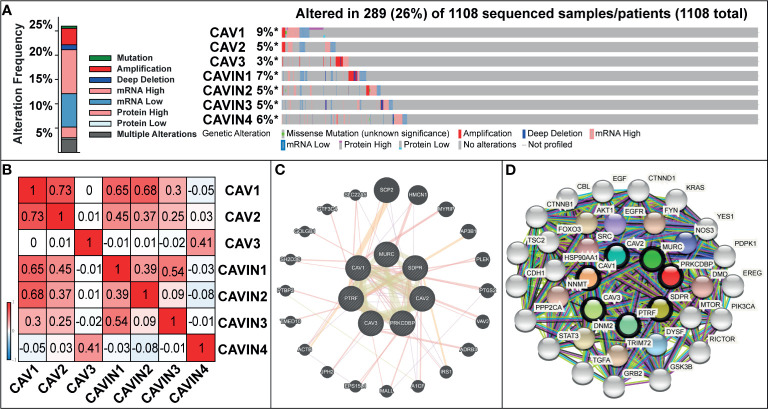
**(A)** Gene mutation analysis of CAVs and CAVINs in patients with breast cancer. **(B)** Pearson’s correction between CAVs and CAVINs. **(C)** GeneMANIA analysis of relevant interactive genes of CAVs and CAVINs. **(D)** Molecular network for CAVs and CAVINs and 37 most frequently altered neighbor genes.

We then used cBioPortal (The Cancer Genome Atlas, Provisional; https://www.cbioportal.org/results/oncoprint?session_id=5e623de7e4b0ff7ef5fd9620) to investigate gene alterations, correlations and networks of CAVs and CAVINs in invasive breast cancer. Results showed that CAVs and CAVINs were altered in 289 samples of 1,108 patients with breast invasive carcinoma (26%) (**Figure 4A**). Among these caveolae-related genes, CAV1 achieved the highest alteration rate at 9%, of which the majority were mRNA dysregulation and amplification (**Figure 4A**). CAVIN1 and CAVIN2 achieved the highest rate of mRNA downregulation, consisting of more than half the alterations (**Figure 4A**). Furthermore, the correlations of CAVs and CAVINs were calculated by analyzing their mRNA expression with the inclusion of Pearson’s correction. The results showed that there were significant and positive correlations in the following CAVs and CAVINs: CAV1 with CAV2, CAVIN1, CAVIN2, and CAVIN3; CAV2 with CAV1, CAVIN1, and CAVIN2; CAV3 with CAVIN4; CAVIN1 with CAV1, CAV2, CAVIN2, and CAVIN3; CAVIN2 with CAV1, CAV2 and CAVIN1; CAVIN3 with CAV1 and CAVIN1; and CAVIN4 with CAV3 (**Figure 4B**). The molecular interaction network was then constructed for CAVs, CAVINs and other most frequently changed neighbor genes with the application of GeneMANIA database (https://genemania.org/) and STRING database (https://string-db.org/cgi/network.pl?taskId=TPhhdLzBayql). In GeneMANIA analysis, results showed strong associations between CAV1, CAVIN1 (PTRF), CAVIN2 (SDPR), and the most prominent weight of SCP2, HMCN1, MYRIP, and AP3B1, suggesting (**Figure 4C**). According to STRING database, our results elucidated that the adhesion-related genes, including SHC1, EGFR, SRC, EGF, FYN, and PI3K-Akt signaling pathway-related genes, including FOXO3, NOS3, EGFR, HSP90AA1, EGF, were significantly associated with caveolins and cavins alterations (**Figure 4D**).

### Relationship Between the Expression of CAVs and CAVINs and Prognosis of Patients With Breast Cancer

Next, we investigated the associations of CAVs and CAVINs levels with survival of the patients. We used Kaplan-Meier Plotter (41) to generate Kaplan Meier survival curve with log rank test under a variety of grouping conditions. Overall survival (OS), relapse-free survival (RFS), distant metastasis-free survival (DMFS), and post progression survival (PPS) were summarized as shown in **Tables 2** **–** **4** and **Figures S1**–**S3**. Results indicated that in all the population of patients with breast cancer in the database, decreased levels of CAV1, CAVIN1 and CAVIN2 were statistically related to poor OS of patients with breast cancer (*p*<0.05) (**Table 2** and **Figure S1**), and lower expression of CAV1, CAV2, CAV3, CAVIN1, CAVIN2, and CAVIN4 were statistically related to shorter relapse-free survival (RFS) of the patients (*p*<0.05) (**Table 2** and **Figure S1**). Among these curves, CAVIN1 and CAVIN2 levels achieved the most remarkable capabilities in distinguishing OS, while CAV3 and CAVIN2 achieved the most in distinguishing RFS.

**Table 2:** Correlation of CAVs/CAVINs transcription levels and survivals of patients with breast cancer.

Gene	Dataset/ Affymetrix ID	Survival outcome	No.of cases	HR	95% CI	*p*-value
CAV1	212097_at	OS	1402	0.81	0.65-1	**0.047**
		RFS	3951	0.87	0.78-0.97	**0.012**
		DMFS	1803	0.91	0.76-1.11	0.36
		PPS	414	0.97	0.76-1.24	0.81
CAV2	203323_at	OS	1402	0.89	0.72-1.1	0.29
		RFS	3951	0.88	0.79-0.98	**0.021**
		DMFS	1803	1.02	0.84-1.23	0.84
		PPS	414	1.08	0.85-1.38	0.52
CAV3	208204_s_at	OS	1402	0.82	0.66-1.01	0.067
		RFS	3951	0.75	0.67-0.84	**2.2E-07**
		DMFS	1803	0.92	0.76-1.11	0.37
		PPS	414	0.87	0.68-1.11	0.25
CAVIN1	208789_at	OS	1402	0.75	0.6-0.92	**0.0073**
		RFS	3951	0.82	0.74-0.91	**0.00035**
		DMFS	1803	1.03	0.85-1.25	0.77
		PPS	414	0.87	0.69-1.11	0.27
CAVIN2	222717_at	OS	626	0.66	0.48-0.9	**0.0087**
		RFS	1764	0.65	0.55-0.76	**4.6E-08**
		DMFS	664	0.78	0.57-1.08	0.14
		PPS	173	1.29	0.91-1.84	0.15
CAVIN3	213010_at	OS	1402	1	0.8-1.23	0.97
		RFS	3951	1.04	0.93-1.16	0.46
		DMFS	1803	1.11	0.92-1.34	0.29
		PPS	414	1.02	0.8-1.3	0.88
CAVIN4	241749_at	OS	626	1.11	0.81-1.51	0.52
		RFS	1764	1.25	1.07-1.46	**0.0045**
		DMFS	664	1.38	1-1.91	0.051
		PPS	173	0.82	0.57-1.17	0.27

Bold values mean p value less than 0.05.

**Table 3:** Correlation of CAVs/CAVINs transcription levels and survivals of patients with different molecular subtypes of breast cancer.

Gene symbol	Survival outcome	Basal		Luminal A		Luminal B		HER2+	
		HR (95% CI)	*p*-value	HR (95% CI)	*p*-value	HR (95% CI)	*p*-value	HR (95% CI)	*p*-value
CAV1	OS	1.71	**0.034**	0.75	0.11	0.82	0.3	1.03	0.94
	RFS	1.46	**0.0034**	0.75	**0.0012**	1.05	0.63	1.38	0.1
	DMFS	1.34	0.24	0.93	0.64	1.12	0.51	1.62	0.13
	PPS	1.62	0.11	1.03	0.89	1.06	0.78	0.66	0.28
CAV2	OS	1.58	0.071	0.76	0.14	0.76	0.15	0.56	0.081
	RFS	1.39	**0.011**	0.79	**0.0061**	0.88	0.18	0.96	0.84
	DMFS	1.46	0.13	0.94	0.67	0.99	0.95	0.97	0.93
	PPS	1.81	**0.049**	1.08	0.68	1.21	0.39	0.65	0.25
CAV3	OS	0.83	0.44	0.65	**0.019**	0.92	0.68	0.6	0.13
	RFS	0.81	0.11	0.77	**0.0025**	0.77	**0.0075**	0.66	**0.036**
	DMFS	0.78	0.32	0.93	0.62	1.11	0.55	0.61	0.12
	PPS	0.6	0.096	0.85	0.42	1.04	0.85	0.99	0.98
CAVIN1	OS	0.72	0.18	0.6	**0.0052**	1.11	0.59	0.88	0.69
	RFS	0.93	0.55	0.71	**0.00009**	0.91	0.32	0.97	0.88
	DMFS	0.9	0.66	0.92	0.55	0.99	0.98	1.99	**0.035**
	PPS	0.95	0.86	0.8	0.27	1.19	0.43	0.95	0.9
CAVIN2	OS	1.33	0.38	0.53	**0.015**	0.63	0.18	0.6	0.21
	RFS	0.99	0.95	0.59	**0.000033**	0.8	0.15	0.78	0.28
	DMFS	0.87	0.69	0.84	0.54	0.95	0.88	0.79	0.53
	PPS	1.13	0.78	1.21	0.51	1	0.99	1.53	0.34
CAVIN3	OS	0.94	0.8	0.88	0.48	1.29	0.18	1.22	0.54
	RFS	1.41	**0.0082**	0.9	0.24	1.13	0.21	1.32	0.16
	DMFS	1.11	0.68	1.08	0.61	1.226	0.19	1.59	0.15
	PPS	0.75	0.34	0.87	0.49	1.38	0.15	0.92	0.82
CAVIN4	OS	0.71	0.29	1.04	0.89	1.19	0.62	1.87	0.13
	RFS	0.88	0.43	1.17	0.21	0.92	0.61	1.66	**0.028**
	DMFS	1.01	0.98	0.95	0.85	1.94	0.051	2	0.073
	PPS	0.51	0.13	0.59	0.075	1.55	0.26	1.23	0.63

Bold values mean p value less than 0.05.

**Table 4:** Correlation of CAVs/CAVINs transcription levels and survivals of patients with different HR/HER2 status of breast cancer.

Gene symbol	Survival outcome	HER2-		PR+		PR-		ER+		ER-	
		HR (95% CI)	*p*-value	HR (95% CI)	*p*-value	HR (95% CI)	*p*-value	HR (95% CI)	*p*-value	HR (95% CI)	*p*-value
CAV1	OS	0.85	0.7	0.96	0.96	1.23	0.67	0.66	**0.021**	2.1	**0.0017**
	RFS	0.97	0.84	0.83	0.3	1.19	0.23	0.81	**0.012**	1.62	**3.20E-05**
	DMFS	1.33	0.52	1.1	0.82	1.67	0.085	0.69	**0.032**	1.89	**0.0042**
	PPS	0.39	0.07	NA	NA	NA	NA	0.7	0.08	1.37	0.24
CAV2	OS	0.97	0.95	0.89	0.86	0.86	0.76	0.64	**0.013**	1.39	0.15
	RFS	0.93	0.57	0.8	0.22	1.26	0.13	0.77	**0.0019**	1.22	0.079
	DMFS	1.32	0.53	1.31	0.52	1.67	0.084	0.69	0.03	1.56	**0.043**
	PPS	1.09	0.87	NA	NA	NA	NA	0.77	0.21	1.4	0.2
CAV3	OS	0.68	0.42	5.12	**0.032**	1.13	0.8	0.85	0.37	0.62	**0.049**
	RFS	1.16	0.26	0.9	0.55	1.13	0.41	0.93	0.41	1.01	0.92
	DMFS	0.93	0.86	1.39	0.44	1.11	0.72	1.29	0.13	0.82	0.35
	PPS	0.76	0.6	NA	NA	NA	NA	1.01	0.96	0.76	0.3
CAVIN1	OS	0.89	0.79	0.34	0.13	1.71	0.26	0.68	**0.034**	0.9	0.65
	RFS	0.93	0.57	0.98	0.93	1.12	0.46	0.85	**0.046**	1.07	0.55
	DMFS	1.06	0.9	1.17	0.7	1.66	0.091	0.84	0.29	1.32	0.21
	PPS	0.54	0.23	NA	NA	NA	NA	0.8	0.29	0.85	0.53
CAVIN2	OS	1.55	0.42	NA	NA	NA	NA	1.11	0.79	1.26	0.52
	RFS	0.52	**2.80E-05**	0.58	**0.006**	0.86	0.42	0.68	**0.01**	1.05	0.77
	DMFS	0.4	0.17	0.34	0.093	1.04	0.92	0.59	0.27	1.07	0.87
	PPS	2.48	0.095	NA	NA	NA	NA	1.68	0.25	1.73	0.18
CAVIN3	OS	1.09	0.84	0.61	0.46	1.35	0.52	1.05	0.79	1.25	0.33
	RFS	1.29	0.056	1.07	0.71	1.5	0.007	0.98	0.79	1.47	**8.60E-04**
	DMFS	0.91	0.84	1.15	0.74	1.41	0.25	0.95	0.78	1.32	0.2
	PPS	0.56	0.26	NA	NA	NA	NA	1.24	0.3	1.01	0.97
CAVIN4	OS	0.66	0.44	NA	NA	NA	NA	0.75	0.46	0.58	0.13
	RFS	1.25	0.14	1.35	0.12	1.14	0.48	1.26	0.12	1.04	0.82
	DMFS	1.11	0.87	0.88	0.83	1.66	0.18	0.84	0.71	1.35	0.48
	PPS	0.84	0.76	NA	NA	NA	NA	1.54	0.35	1.03	0.94

Bold values mean p value less than 0.05.

Then we investigated whether CAVs and CAVINs levels significantly affected prognosis of patients with different molecular subtypes of breast cancer. Our results elucidated that lower expression of CAV3, CAVIN1, and CAVIN2 significantly related to OS of patients with Luminal A breast cancer (**Table 3** and **Figure S2**). Protein levels of all CAVs, CAVIN1, and CAVIN2 were associated with RFS of patients with Luminal A breast cancer, suggesting these caveolae-related genes can be potentially independent predictive factors of the prognosis of Luminal A breast cancer (**Table 3** and **Figure S2**). Remarkably, lower expression levels of CAV1, CAV2, and CAVIN3 were significantly relevant to better RFS of patients with basal-like breast cancer, indicating in triple-negative breast cancer, caveolae-related genes may play different roles in carcinogenesis and tumor progression as them in hormone receptor-positive breast cancer (**Table 3** and **Figure S2**).

Furthermore, the associations between protein levels and survival of patients was the most obvious in the KM curves revealing the expression of CAVIN2 and RFS of HER2 negative, progesterone receptor (PR) positive, and estrogen receptor (ER) positive breast cancer (**Table 4** and **Figure S3**). CAV1 and CAV2 also elucidated independent predicting value in the RFS of ER+ breast cancer patients, but the prognosis of breast cancer patients with other receptor status was not well differentiated by expression levels of CAV1 and CAV2 (**Table 4** and **Figure S3**). In conclusion, CAVIN2 achieved the highest potential of independently predicting prognosis of patients with ER+ breast cancer among all the caveolae-related genes.

Based on the above investigations, we further cross-analyzed the RFS of patients with breast cancer for intra-family study. The comparison within CAV family showed that no matter how much CAVs expressed, the transcriptional level of CAV3 can always distinguish RFS of patients with statistically differences, and the similar situation occurred on CAVIN2 from CAVINs family as shown in **Figure S4**. Taken together, these results revealed a remarkable prognostic potential of CAVIN2 in breast cancer populations.

### Prediction of Functions and Related Signaling Pathway of CAVs and CAVINs in Patients With Breast Cancer

**Figure 5:**
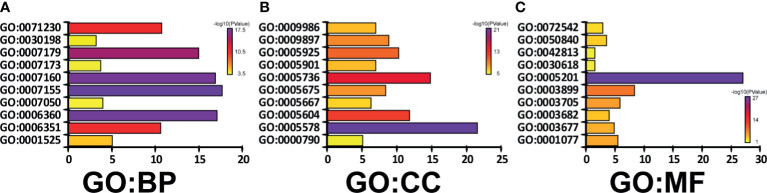
Genes significantly associated with CAVs and CAVINs alterations indicated by GO enrichment as **(A)** biological processes, **(B)** cellular components, and **(C)** molecular functions.

To predict the function of CAVs and CAVINs and the genes significantly related to the alterations of them, we investigated the gene ontology (GO) and Kyoto Encyclopedia of Genes and Genomes (KEGG) in the Database for Annotation, Visualization and Integrated Discovery (DAVID) (https://david.ncifcrf.gov/summary.jsp). In GO enrichment analysis, we can obtain prediction of the functional roles of these genes on biological processes, cellular components, and molecular functions. Our results showed that GO:0007155 (cell adhesion), GO:0007160 (cell-matrix adhesion), GO:0006360 (transcription from RNA polymerase I promoter), and GO:0007179 (transforming growth factor beta receptor signaling pathway) were significantly regulated by CAVs and CAVINs alterations in breast adenocarcinoma (**Figure 5A**). GO:0005578 (proteinaceous extracellular matrix) and GO:0005201 (extracellular matrix structural constituent) were significantly influenced by alterations of caveolae-related genes (**Figures 5B, C**). These results indicated that the major function of CAVs and CAVINs mainly associate with cell surface components and their related molecular pathways.

**Figure 6:**
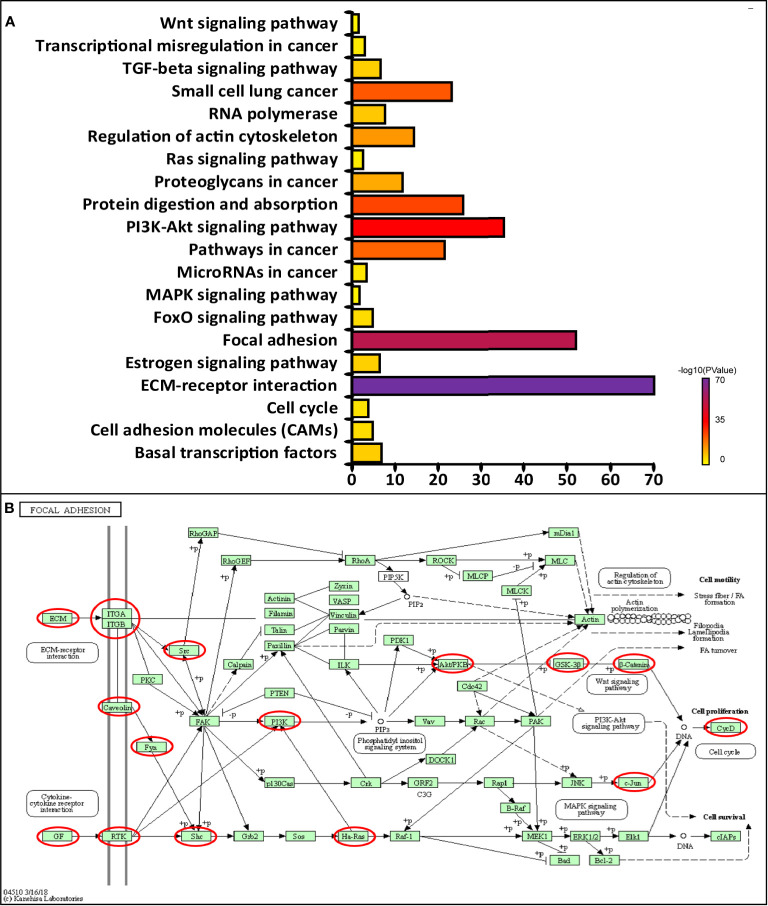
**(A)** Molecular pathways associated with CAVs and CAVINs regulation implemented by DAVID tools. **(B)** Focal adhesion pathways regulated by CAVs and CAVINs in breast cancer.

Next, we conducted KEGG analysis to explore the pathways related to CAVs and CAVINs altered functions and the frequently altered neighbor genes. The results showed 20 pathways related to the functions of CAVs and CAVINs alterations in breast adenocarcinoma through (**Figure 6A**). Among these pathways, cfa04512: ECM-receptor interaction, cfa04510: focal adhesion, cfa04151: PI3K-Akt signaling pathway, and cfa05200: Pathways in cancer were prominently involved in the tumorigenesis and progression of breast cancer (**Figures 6A, B**).

**Figure 7:**
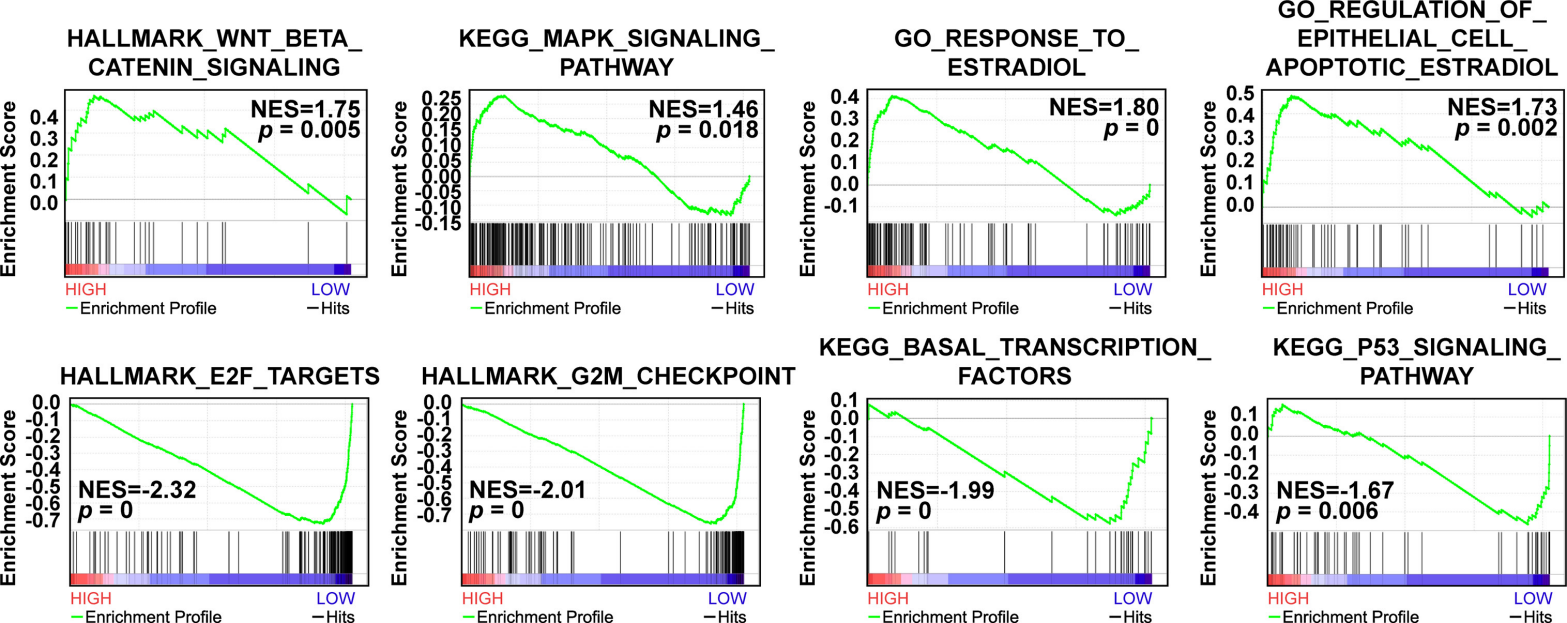
GSEA analysis of altered CAVIN2 expression levels and relevant molecular pathways.

Based on the survival results, we then implemented Gene Set Enrichment Analysis (GSEA) analysis to explore the relevant signaling pathways of CAVIN2 high expression group and low expression group of patients with breast cancer patients. Our results revealed significant relations between the expressions of several cancer related signaling pathways such as Wnt/β-catenin signaling pathway, MAPK signaling pathway, E2F targets, G2M checkpoint components, and others related genes in breast carcinoma (**Figure 7**). The highly enriched gene sets in high expression group included Wnt/β-catenin signaling pathway (NES=1.75, *p*=0.005), MAPK signaling pathway (NES=1.46, *p*=0.018), response to estradiol (NES=1.80, *p*=0), and regulation of estradiol on apoptosis of epithelial cells (NES=1.73, *p*=0.002). In low expression group, the highly enriched gene sets included E2F targets (NES=-2.32, *p*=0), G2M checkpoints (NES=-2.01, *p*=0), transcription factors sets in basal-like (NES=-1.99, *p*=0), and P53 signaling pathway (NES=-1.67, *p*=0.006) (**Figure 7**).

## Discussion

Dysregulations of CAVs and CAVINs have been studied in many diseases, but few of them focused on the tumorigenesis, progression, and prognosis of cancer. Our study is the first to comprehensively investigate the expressions and prognosis of all caveolae-related proteins in patients with breast cancer. These findings are expected to provide solid evidences for target therapy towards breast cancer in the future, from which patients can obtain better outcomes and longer survival durations.

According to the genomic sequencing results from several investigated database, caveolae-related proteins all exhibited downregulation in breast cancer tissues except for CAVIN3. This indicated that most of CAVs and CAVINs acted as tumor suppressors in breast cancer development. However, we can conclude from Oncomine results that only CAVIN2 were downregulated in most types of cancers, while the expression levels of others were significantly elevated in specific types of cancers such as kidney cancer and lymphoma. From this prospective, CAV1, CAV2, and CAVIN1 can also play important roles in oncogenesis. The dual role of these proteins will need more investigations focusing organ-specific tumorigenesis in the future.

In terms of genes that code caveolins, CAV1 have been studied the most. Our investigation revealed that it was statistically less expressed in breast cancer tissues, which was elucidated by several databases as well as our immunohistochemistry results. It can be also correlated with OS and RFS of patients with breast cancer. The alteration rate in breast cancer was the highest among all the caveolae-related genes. Considering CAV1’s association with breast cancer stem cell enrichment, these results implied it as a potential predictor of breast cancer progression. But as the most essential component of caveolae structure, the molecular mechanisms underlying the relationship between its downregulation and poor prognosis of patients with breast cancer need to be further understood.

Comparatively, CAV2 and CAV3 were less studied in cancer, but CAV3 have showed a better potential in correlating to breast cancer development. As a sub-structure of caveolae, CAV3 only revealed association with CAVIN4, which were both little expressed in cancer cells. Although intrafamily cross-analysis of survival curves indicated that CAV3 could be an independent predictor in regardless of the expression levels of other CAVs (**Figure S4**), evidences should be further consolidated to indicate whether it could accurately evaluate the risk of patients with breast cancer after receiving treatments.

The comprehensive analysis of cavins’ expressions and functions had provided evidences of significant roles of CAVIN1 and CAVIN2 in inhibiting breast cancer development. Both CAVIN1 and CAVIN2 were significantly downregulated in breast cancer tissues and were associated with prognosis of patients. Previous studies have reported that loss of CAVIN1 induced a reduction in numbers of caveolae (32), and CAVIN1 knockout mice demonstrated a lack of caveolae, glucose intolerance and disorders in lungs and cardiovascular system (51–53). As a consequence, therapeutic methods targeting CAVIN1 may induce complications in cancer patients. Compared to CAVIN1, CAVIN2 demonstrated a better potential to serve as a therapeutic target. Downregulation of CAVIN2 in breast cancer tissues was the most significant of all caveolae-related genes, and it could assess prognostic values of patients with breast cancer in regardless of all others CAVINs (**Figure S4**). As previous studies have proved that downregulation of CAVIN2 could cause reduction in CAVIN1 and CAV1 expression (54), the interdependency of these 3 molecules makes CAVIN2 as a prominent therapeutic target in breast cancer treatment.

Furthermore, an important question raised from our current study is that which is more accurate in terms of results concluded from public database and real-world experiments. As in **Figure 1**, the results concluded from Oncomine database were not based on paired samples from patients with breast cancer. While in **Figure 3**, we have presented the most representative images for IHC experiments, which were derived from 20 paired samples from patients with breast cancer. Based on our current knowledge, Oncomine analysis and TCGA data just represent an average level of transcription or protein expression, which should be less accurate than real-world validation using paired samples. However, the amount of samples we used for IHC was not enough to compare with the very large scale study as public data. As a consequence, we believe that the Oncomine analysis and TCGA data should be more accurate in our current work. In our future work, multiple cohorts of samples from different hospitals at a larger amount will be considered to validate public data as well as the results presented by our experiments.

Meanwhile, predictions on neighbouring pathways of caveolae-related genes implied that the locations of CAVs and CAVINs were closely relevant to their functions, as the altered levels of these genes mainly influence molecules and pathways involved in cell adhesion and extracellular matrix function. Future studies can focus on the interactions between CAVs, CAVINs, proteins of extracellular matrix, and cell surface receptors, to further uncover the association of caveolae-related genes to the biological properties of cancer cells.

In conclusion, in this study we comprehensively analyzed the expression levels and prognostic values of caveolae-related genes in breast cancer, and provided an overview of their related molecular pathways and biological properties by investigating genomic sequencing data in several online databases. These results elucidated that most of CAVs and CAVINs act as suppressors in breast cancer tumorigenesis, and CAVIN2 were closely related to evaluating the risk of patients with breast cancer. Our findings suggested CAVIN2 could be potential targets for breast cancer therapy. Its downregulation could be a promising predictor for assessing prognostic values of breast cancer.

## Data Availability Statement

The original contributions presented in the study are included in the article/**Supplementary Material**. Further inquiries can be directed to the corresponding authors.

## Ethics Statement

The studies involving human participants were reviewed and approved by Institutional Review Board of Tianjin Medical University Cancer Institute and Hospital. Written informed consent for participation was not required for this study in accordance with the national legislation and the institutional requirements.

## Author Contributions

Conceptualization, YT. Investigation, YT, XL, and YY. Writing – Original Draft, YT and XL. Writing – Review & Editing, YT, XL, JH, HZ, BW, YL, LF, RS, and YY. Visualization, YT, XL, RS, and YY. Supervision, RS and YY. Funding Acquisition, XL and YY. All authors contributed to the article and approved the submitted version.

## Funding

This study was funded by the National Natural Science Foundation of China (No. 82172827 and 81502518) and Tianjin Bureau of Foreign Experts Affairs JH-20180070802-2018038.

## Conflict of Interest

The authors declare that the research was conducted in the absence of any commercial or financial relationships that could be construed as a potential conflict of interest.

The reviewer XZ declared a shared affiliation, with no collaboration, with the authors to the handling editor at the time of the review. 

## Publisher’s Note

All claims expressed in this article are solely those of the authors and do not necessarily represent those of their affiliated organizations, or those of the publisher, the editors and the reviewers. Any product that may be evaluated in this article, or claim that may be made by its manufacturer, is not guaranteed or endorsed by the publisher.

## Supplementary Material

Supplementary Figure 1 Overall survival (OS), relapse-free survival (RFS), distant metastasis-free survival (DMFS), and post progression survival (PPS) curves of patients with breast cancer under different expression levels of CAVs and CAVINs.

Supplementary Figure 2 OS, RFS, DMFS, and PPS curves of patients with different molecular subtypes of breast cancer divided by different expression levels of CAVs and CAVINs.

Supplementary Figure 3 OS, RFS, DMFS, and PPS curves of patients with different receptor status of breast cancer divided by different expression levels of CAVs and CAVINs.

Supplementary Figure 4 Intra-family cross analysis of different CAVs and CAVINs levels on RFS of patients with breast cancer.
