# Supplementary figures and images for: Integrated Bioinformatic Analysis of the Expression and Prognosis of Caveolae-Related Genes in Human Breast Cancer
Source: Front Oncol. 2021 Aug 26;11:703501. doi: 10.3389/fonc.2021.703501 (PMC8427033; doi:10.3389/fonc.2021.703501)

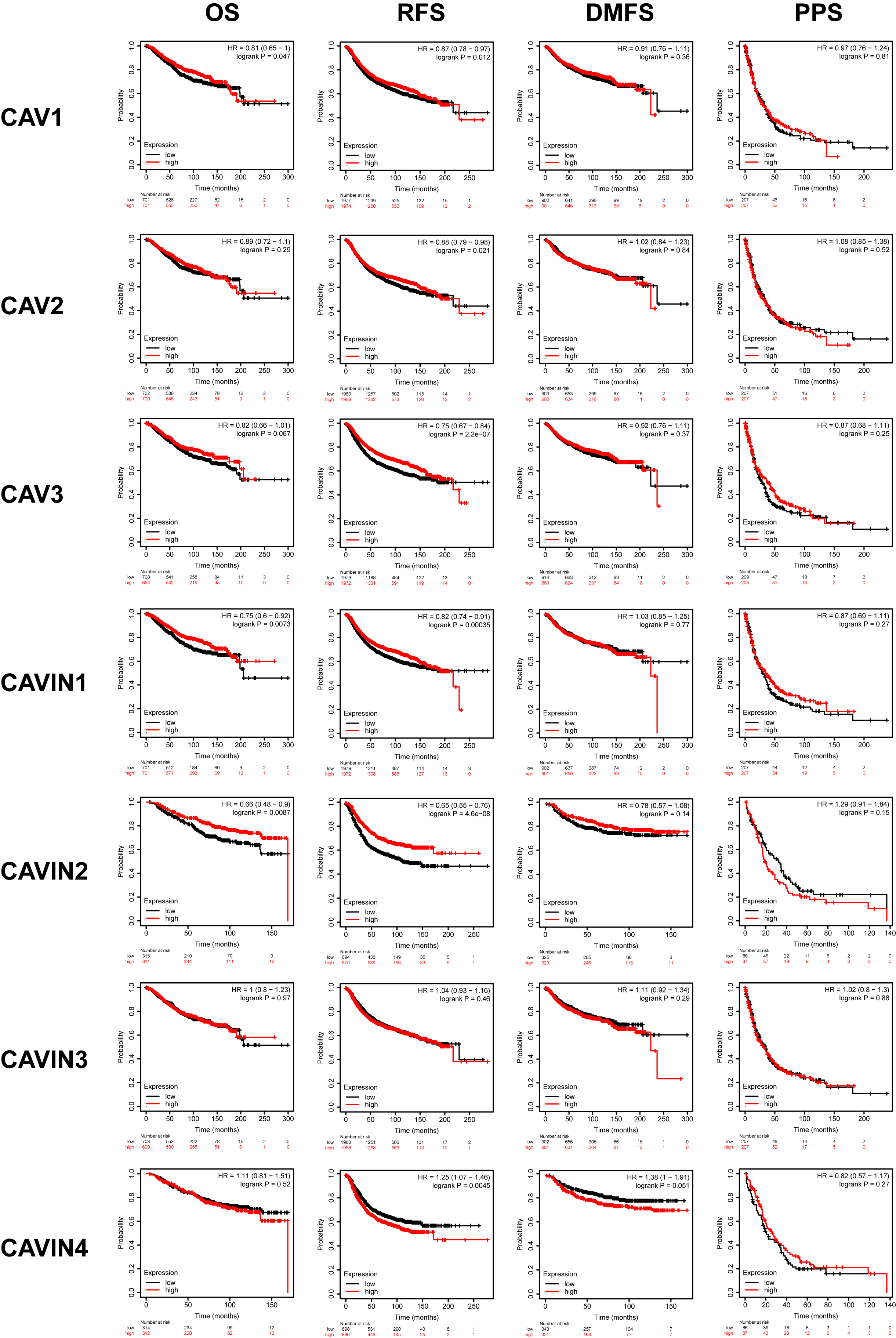

Supplement: Supplementary Figure 1 — Overall survival (OS), relapse-free survival (RFS), distant metastasis-free survival (DMFS), and post progression survival (PPS) curves of patients with breast cancer under different expression levels of CAVs and CAVINs. [file Image_1.tif]

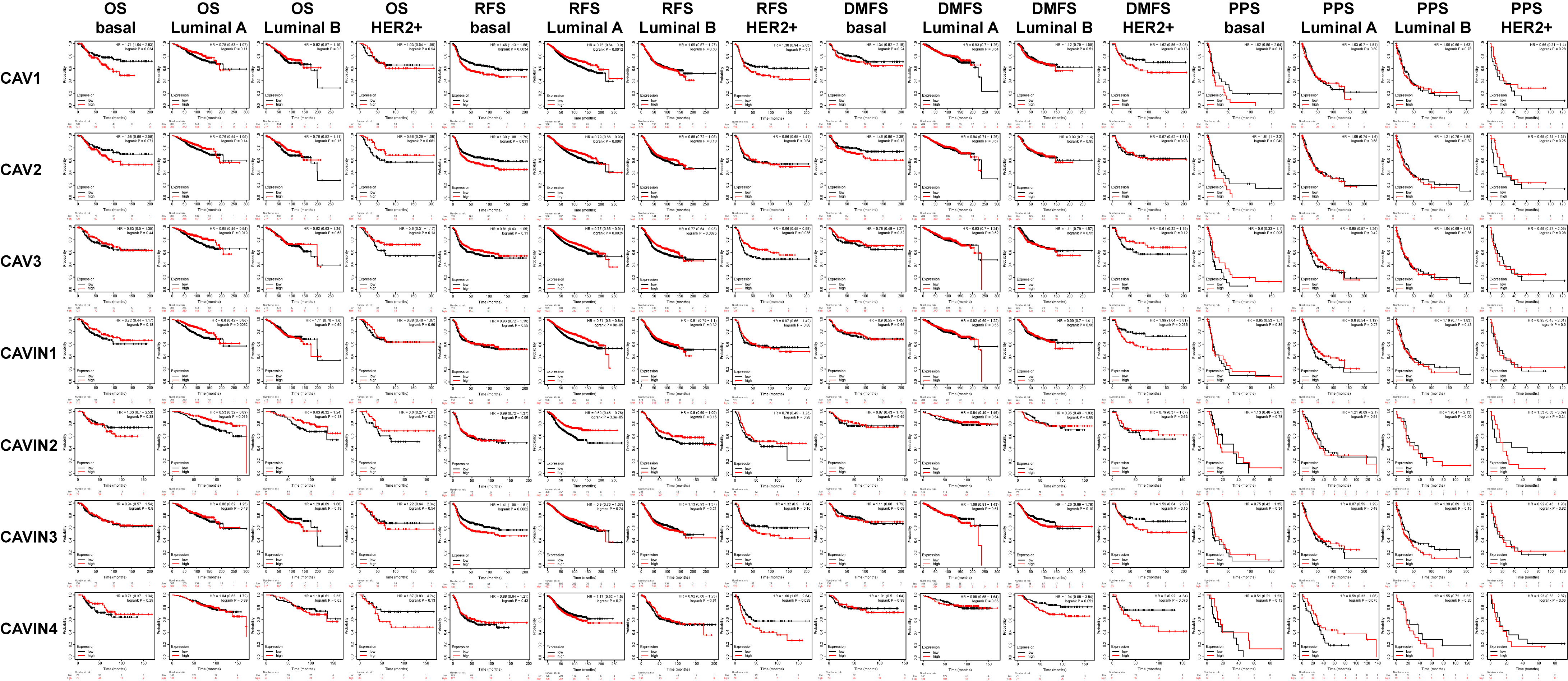

Supplement: Supplementary Figure 2 — OS, RFS, DMFS, and PPS curves of patients with different molecular subtypes of breast cancer divided by different expression levels of CAVs and CAVINs. [file Image_2.tif]

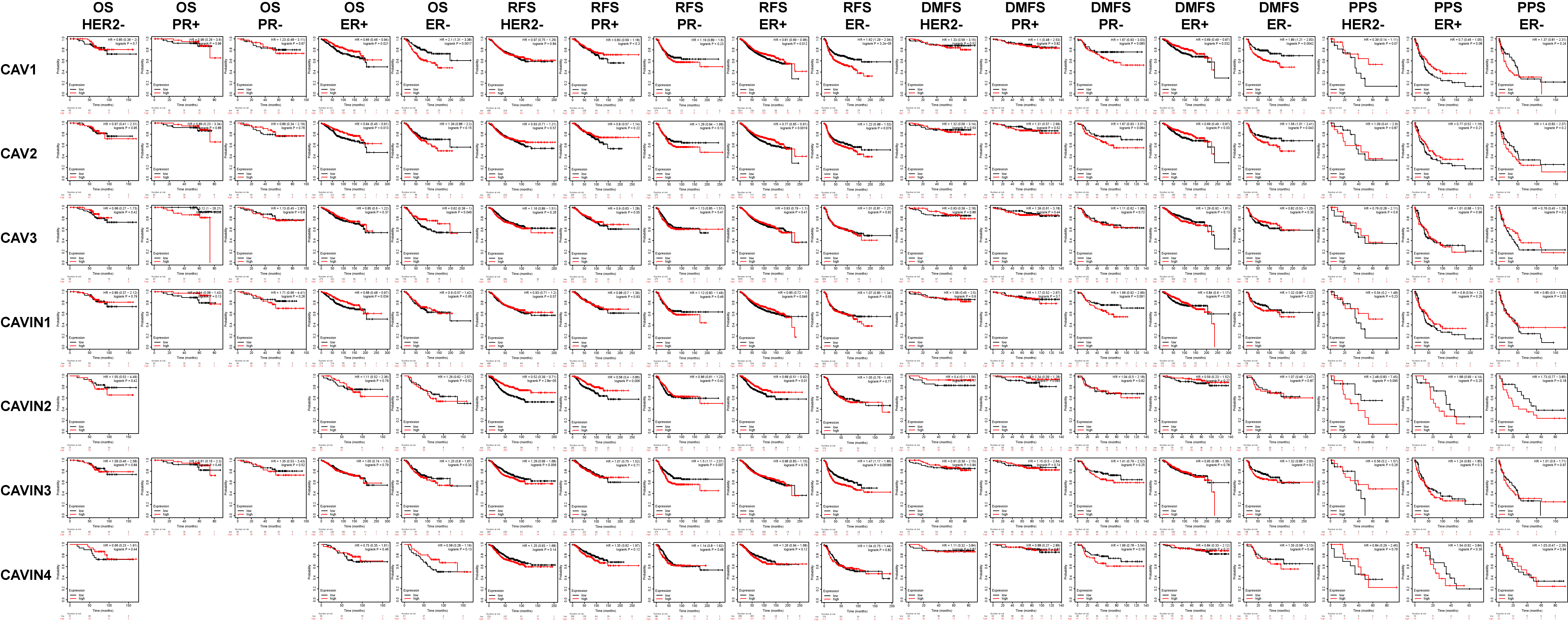

Supplement: Supplementary Figure 3 — OS, RFS, DMFS, and PPS curves of patients with different receptor status of breast cancer divided by different expression levels of CAVs and CAVINs. [file Image_3.tif]

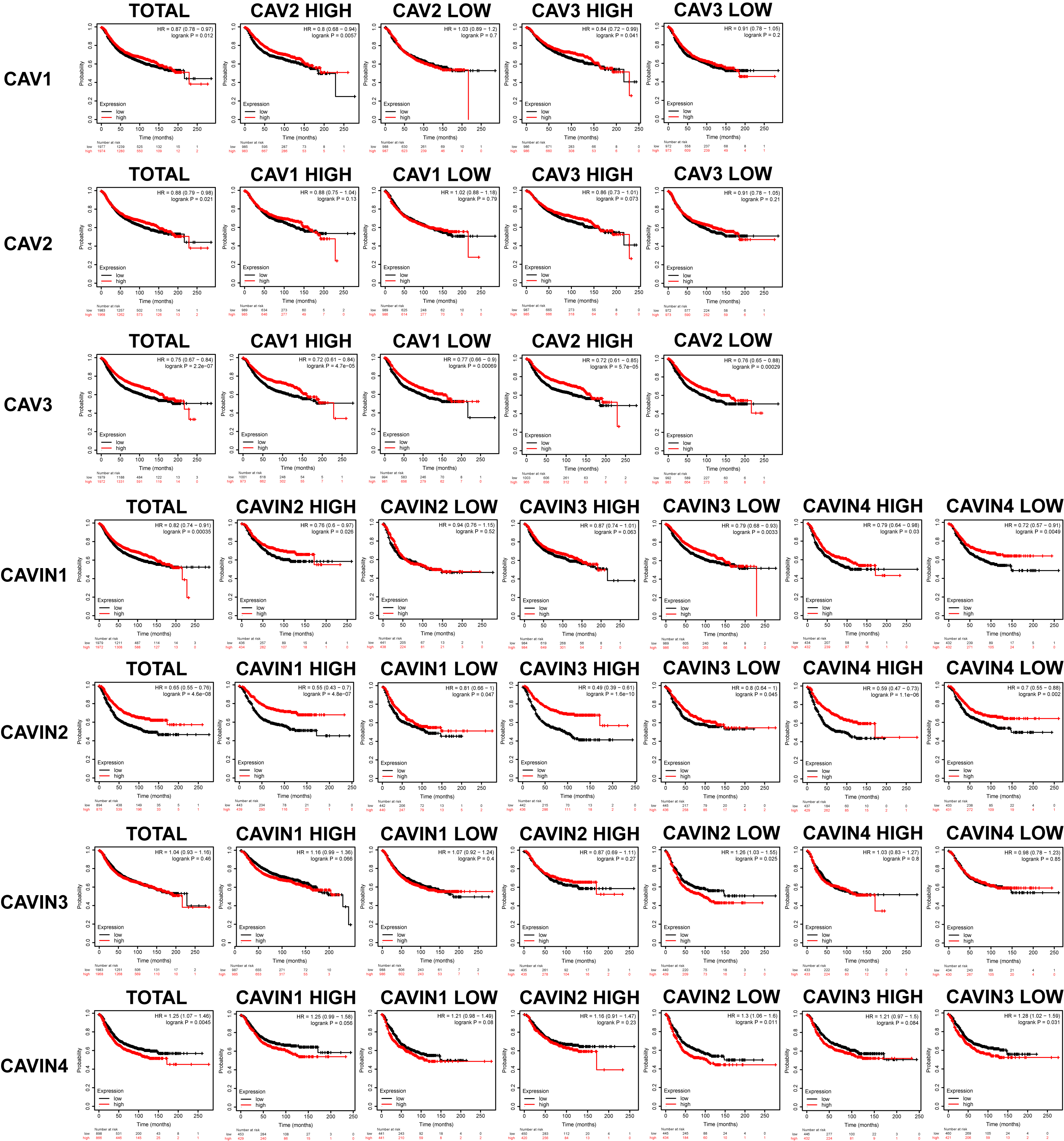

Supplement: Supplementary Figure 4 — Intra-family cross analysis of different CAVs and CAVINs levels on RFS of patients with breast cancer. [file Image_4.tif]
